# The thalamostriatal system in normal and diseased states

**DOI:** 10.3389/fnsys.2014.00005

**Published:** 2014-01-30

**Authors:** Yoland Smith, Adriana Galvan, Tommas J. Ellender, Natalie Doig, Rosa M. Villalba, Icnelia Huerta-Ocampo, Thomas Wichmann, J. Paul Bolam

**Affiliations:** ^1^Yerkes National Primate Research Center, Emory UniversityAtlanta, GA, USA; ^2^Department of Neurology, Emory UniversityAtlanta, GA, USA; ^3^Udall Center of Excellence for Parkinson’s Disease, Emory UniversityAtlanta, GA, USA; ^4^Department of Pharmacology, MRC Anatomical Neuropharmacology UnitOxford, UK

**Keywords:** thalamus, Parkinson’s disease, intralaminar nuclei, glutamate, vesicular glutamate transporter, attention, striatum, Tourette’s syndrome

## Abstract

Because of our limited knowledge of the functional role of the thalamostriatal system, this massive network is often ignored in models of the pathophysiology of brain disorders of basal ganglia origin, such as Parkinson’s disease (PD). However, over the past decade, significant advances have led to a deeper understanding of the anatomical, electrophysiological, behavioral and pathological aspects of the thalamostriatal system. The cloning of the vesicular glutamate transporters 1 and 2 (vGluT1 and vGluT2) has provided powerful tools to differentiate thalamostriatal from corticostriatal glutamatergic terminals, allowing us to carry out comparative studies of the synaptology and plasticity of these two systems in normal and pathological conditions. Findings from these studies have led to the recognition of two thalamostriatal systems, based on their differential origin from the caudal intralaminar nuclear group, the center median/parafascicular (CM/Pf) complex, or other thalamic nuclei. The recent use of optogenetic methods supports this model of the organization of the thalamostriatal systems, showing differences in functionality and glutamate receptor localization at thalamostriatal synapses from Pf and other thalamic nuclei. At the functional level, evidence largely gathered from thalamic recordings in awake monkeys strongly suggests that the thalamostriatal system from the CM/Pf is involved in regulating alertness and switching behaviors. Importantly, there is evidence that the caudal intralaminar nuclei and their axonal projections to the striatum partly degenerate in PD and that CM/Pf deep brain stimulation (DBS) may be therapeutically useful in several movement disorders.

## Introduction

Although the evolution of the thalamus and striatum pre-dates the expansion of the cerebral cortex (Butler, 1994; Reiner, 2010; Stephenson-Jones et al., 2011), our knowledge about the functional anatomy and behavioral role of the connections between them is minimal compared to the amount of information that has been gathered about the corticostriatal system (Kemp and Powell, 1971; Alexander et al., 1986; Parent and Hazrati, 1995). However, significant progress has been made in our understanding of the anatomical and functional organization of the thalamostriatal system since its first description in the early 1940’s (Vogt and Vogt, 1941; Cowan and Powell, 1956). Research in the last decade has resulted in a much better understanding of various aspects of the synaptic properties of the thalamostriatal projection(s), and their potential roles in cognition. Furthermore, evidence that the center median/parafascicular (CM/Pf) complex, the main source of thalamostriatal connections, is severely degenerated in Parkinson’s disease (PD), combined with the fact that lesion or deep brain stimulation (DBS) of this nuclear group alleviates some of the motor and non-motor symptoms of Tourette’s syndrome (TS) and PD, has generated significant interest in these projections. In this review, we will discuss these topics and provide an overview of our current knowledge of the functional anatomy, synaptology, and physiology of the mammalian thalamostriatal system, as well as the involvement of these projections in disease processes. Because of space limitation, we will focus mostly on recent developments in this field. Readers are referred to previous reviews for additional information and a broader coverage of the early literature on the thalamostriatal projections (Groenewegen and Berendse, 1994; Parent and Hazrati, 1995; Mengual et al., 1999; Haber and Mcfarland, 2001; Van der Werf et al., 2002; Kimura et al., 2004; Smith et al., 2004, 2009, 2010, 2011; McHaffie et al., 2005; Haber and Calzavara, 2009; Halliday, 2009; Minamimoto et al., 2009; Sadikot and Rymar, 2009; Galvan and Smith, 2011; Bradfield et al., 2013b).

## Thalamostriatal circuitry and synaptic connectivity

### Functionally segregated basal ganglia-thalamostriatal circuits through the center median/parafascicular (CM/Pf) complex

**Figure 1:**
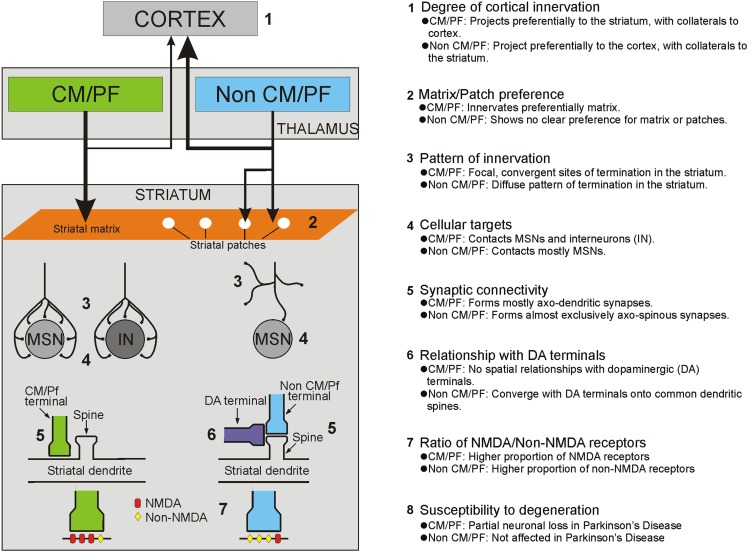
**Summary of the anatomical, functional and pathological characteristics that differentiate thalamostriatal projections from the CM/Pf vs. other thalamic nuclei (i.e., non CM/Pf)**.

Although the thalamostriatal system originates from most thalamic nuclei, the CM/Pf (or the Pf in rodents) is the main source of thalamostriatal projections in primates and non-primates (Smith and Parent, 1986; Berendse and Groenewegen, 1990; Francois et al., 1991; Sadikot et al., 1992a,b; Deschenes et al., 1996a,b; Mengual et al., 1999; McFarland and Haber, 2000; Mcfarland and Haber, 2001; Smith et al., 2004, 2009; Castle et al., 2005; McHaffie et al., 2005; Parent and Parent, 2005; Raju et al., 2006; Lacey et al., 2007). CM/Pf neurons send massive and topographically organized projections to specific regions of the dorsal striatum, but provide only minor inputs to the cerebral cortex (Sadikot et al., 1992a; Parent and Parent, 2005; Galvan and Smith, 2011; Figure 1). Single cell tracing studies in monkeys have shown that more than half of all CM neurons innervate densely and focally the striatum without significant input to the cerebral cortex, while about one third innervates diffusely the cerebral cortex without significant projections to the striatum, and the remaining neurons project to both targets with a preponderance of innervation of the dorsal striatum (Parent and Parent, 2005).

Based on its preferential targeting of specific functional territories, the primate CM/Pf complex is divided into five major sub-regions: (1) the rostral third of Pf which innervates mainly the nucleus accumbens; (2) the caudal two thirds of Pf which project to the caudate nucleus; (3) the dorsolateral extension of Pf (Pfdl) which targets selectively the anterior putamen; (4) the medial two thirds of CM (CMm) which innervates the post-commissural putamen; and (5) the lateral third of CM (CMl) which is the source of inputs the primary motor cortex (M1). Through these projections, the CM/Pf gains access to the entire striatal complex, thereby making the CM/Pf-striatal system a functionally organized network that may broadly affect motor and non-motor basal ganglia functions (Smith et al., 2004, 2009, 2010; Galvan and Smith, 2011). In rodents, the lateral part of Pf is considered to be the homologue of the primate CM and projects mainly to the sensorimotor region (i.e., the dorsolateral part) of the caudate-putamen complex, whereas the medial rodent Pf displays strong similarities with the primate Pf, projecting to associative and limbic striatal regions of the striatum (Groenewegen and Berendse, 1994).

### Thalamostriatal systems from non-center median/parafascicular (CM/Pf) thalamic nuclei

In addition to the CM/Pf complex, thalamostriatal projections originate from several other rostral intralaminar and non-intralaminar thalamic nuclei. In primates and non-primates, the rostral intralaminar nuclei (central lateral, paracentral, central medial), the mediodorsal nucleus (MD), the pulvinar, the lateral posterior nucleus, the medial posterior nucleus, midline, anterior and the ventral motor nuclear group are prominent sources of thalamostriatal projections to the caudate nucleus and putamen (Royce, 1978; Beckstead, 1984; Smith and Parent, 1986; Groenewegen and Berendse, 1994; Smith et al., 2004, 2009; Alloway et al., 2014). In contrast to the projections from the CM/Pf complex, these nuclei send major projections to the cerebral cortex, while contributing a modest or sparse innervation of the dorsal and ventral striatum (Royce, 1983; Macchi et al., 1984; Deschenes et al., 1996a,b; Smith et al., 2004, 2009; Figure 1). In rats, the topography of these projections corresponds to the functionally segregated organization of the striatum, so that sensorimotor-, associative- and limbic-related thalamic nuclei innervate functionally corresponding regions of the dorsal and ventral striatum (Berendse and Groenewegen, 1990, 1991; Groenewegen and Berendse, 1994). Although such detailed analyses have not been done in primates (except for projections from the ventral anterior/ventral lateral (VA/VL) complex), evidence from retrograde and anterograde labeling studies indicate that the primate non-CM/Pf thalamostriatal projections also display a strict functional topography (Parent et al., 1983; Smith and Parent, 1986; Fenelon et al., 1991; McFarland and Haber, 2000; Mcfarland and Haber, 2001). The thalamostriatal projections from the ventral motor thalamic nuclei have received particular attention in these studies. It appears that projections from the pars oralis of the VL (VLo), the main recipient of sensorimotor internal globus pallidus (GPi) outflow, terminate preferentially in the post-commissural putamen, whereas projections from the magnocellular division of the VA, the principal target of the substantia nigra pars reticulata (SNr) and associative GPi outflow, innervate the caudate nucleus (Mcfarland and Haber, 2001). Within striatal territories, VA/VL projections terminate in a patchy manner in the striatum, indicating that additional organizational principles may be at work that have not yet been elucidated (Groenewegen and Berendse, 1994; McFarland and Haber, 2000; Mcfarland and Haber, 2001; Smith et al., 2004, 2009; Raju et al., 2006).

Through the use of trans-synaptic viral tracing studies, Strick and colleagues have suggested that thalamostriatal projections from the VL and other intralaminar thalamic nuclei that receive cerebellar outflow from the dentate nucleus may be the underlying connections through which the cerebellum communicates with the basal ganglia (Bostan and Strick, 2010; Bostan et al., 2010, 2013). Dysfunctions of these cerebello-thalamo-basal ganglia interactions may underlie some aspects of the pathophysiology of dystonia and other movement disorders (Jinnah and Hess, 2006; Neychev et al., 2008; Calderon et al., 2011).

### The superior colliculus: A potential driver of the thalamostriatal system in mammals

Anatomical studies have suggested that additional sub-cortical tecto-basal ganglia loops exist that connect the superficial and deep layers of the superior colliculus with specific thalamic nuclei, which then gain access to the basal ganglia circuitry via thalamostriatal connections (McHaffie et al., 2005; Redgrave et al., 2010). The existence of these connections could resolve some of the fundamental issues associated with short-latency responses to biologically salient stimuli (Smith et al., 2011; Alloway et al., 2014). As discussed in more detail below, Pf neurons exhibit short-latency excitatory responses to salient stimuli. Redgrave and colleagues have suggested that the superior colliculus (optic tectum in lower species) displays the evolutionary profile, anatomical connectivity and physiological features that would allow it to mediate such effects upon thalamic neurons (McHaffie et al., 2005; Redgrave et al., 2010). The basal ganglia and the superior colliculus are neural structures that appeared early (>400 million years ago) and have been highly conserved throughout the evolution of the vertebrate brain (Reiner, 2010; Stephenson-Jones et al., 2011), thereby suggesting that they are part of fundamental processing units that play basic functions in mammalian behavior. The superior colliculus has direct access to primary sensory information, and studies have shown that stimuli associated with positive or negative outcomes activate different sub-regions of this nucleus that engage various tecto-thalamo-striatal loops (Redgrave et al., 1999; Alloway et al., 2014). Through these loops, the primary sensory events could be rapidly transmitted to the striatum and affect the basal ganglia circuitry which, in turn, could lead to basal ganglia-mediated disinhibition of different sub-regions of the superior colliculus that could help select and reinforce some sensory stimuli over others (Redgrave et al., 1999), most likely through regulation of corticostriatal plasticity via dopaminergic and cholinergic intrastriatal mechanisms (Ding et al., 2010; Smith et al., 2011). Through these processes, the short-latency sensory-driven activity in the superior colliculus could be used by the basal ganglia to reinforce the development of novel habits or procedures (Redgrave et al., 2010; Smith et al., 2011).

### Synaptic organization and prevalence of thalamostriatal vs. corticostriatal terminals

**Figure 2:**
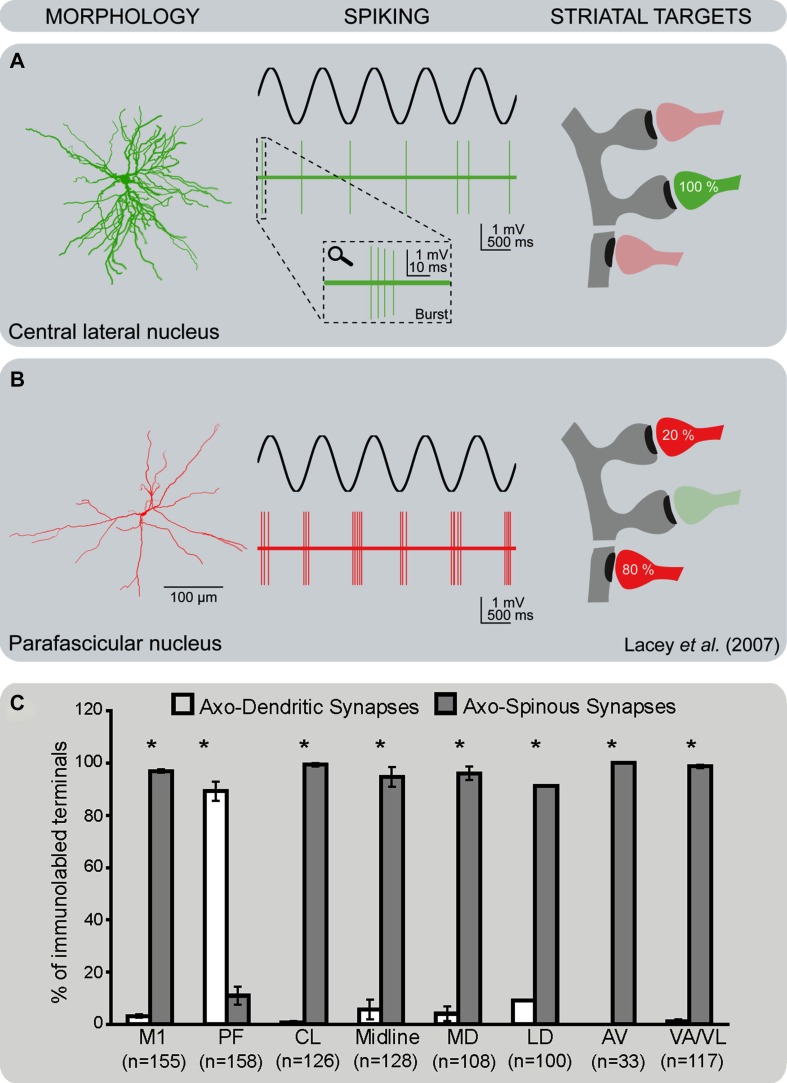
**Differential pattern of activity and synaptic connectivity of CM/Pf vs. non-CM/Pf thalamostriatal terminals. (A)** Thalamostriatal neurons of the central lateral nucleus (non-CM/Pf) have a compact bushy dendritic arbor. They tend to fire brief bursts of spikes at high frequency (2–5 spikes at ~150 Hz) in time with the active part of the cortical slow oscillation, reminiscent of low-threshold Ca^2+^ spike bursts. At the ultrastructural level, terminals from the central lateral nucleus almost exclusively target spines in the striatum. **(B)** Thalamostriatal neurons of the parafascicular nucleus have a reticular dendritic arbor with long and infrequently branching dendrites. They also tend to fire bursts of action potentials in time with the active part of the cortical slow oscillation, but the burst spike frequency is significantly lower and more variable than those of central lateral neurons. At the ultrastructural level, terminals from the parafascicular nucleus predominantly target the dendritic shafts of striatal neurons (see Lacey et al., 2005, 2007 for more details). **(C)** Histogram showing the frequency of axo-dendritic vs. axo-spinous synapses formed by anterogradely labeled boutons from various thalamic nuclei and the primary motor cortex (M1) in the rat striatum. Note the striking difference in the pattern of synaptic connectivity of terminals from Pf vs. other thalamic nuclei (see Raju et al., 2006 for more details). Abbreviations: AV: Anteroventral nucleus; CL: Central lateral nucleus; LD: Laterodorsal nucleus; MD: Mediodorsal nucleus; M1: Primary motor cortex; Pf: Parafascicular nucleus; VA/VL: Ventral anterior/ventral lateral nucleus.

Anterograde tracing studies in several species have shown that the thalamostriatal projections give rise to asymmetric (or Gray’s Type 1) synapses. In rodents, the principal synaptic target of most non-CM/Pf thalamostriatal projections are dendritic spines of striatal medium spiny neurons (MSNs), a pattern of synaptic connectivity similar to the corticostriatal system (Kemp and Powell, 1971; Dube et al., 1988; Xu et al., 1991; Raju et al., 2006; Lacey et al., 2007; Figures 1, 2). In contrast, striatal afferents from CM/Pf (or Pf in rodents) establish asymmetric synapses principally with dendritic shafts of MSNs (Dube et al., 1988; Sadikot et al., 1992b; Smith et al., 1994; Sidibe and Smith, 1996; Raju et al., 2006, 2008; Lacey et al., 2007) and several types of striatal interneurons including cholinergic interneurons (Meredith and Wouterlood, 1990; Lapper and Bolam, 1992; Sidibe and Smith, 1999) and parvalbumin-positive GABA interneurons (Rudkin and Sadikot, 1999; Sidibe and Smith, 1999; Figure 1). Overall, 70–90% of CM/Pf (or Pf) terminals form axo-dendritic synapses in the rat and monkey striatum (Dube et al., 1988; Sadikot et al., 1992b; Raju et al., 2006; Lacey et al., 2007). However, single cell filling studies have revealed that the pattern of synaptic connection of individual Pf neurons is highly variable in rats. For instance, some Pf neurons were found to be the sources of terminals that terminate almost exclusively on dendritic spines, whereas others predominantly target dendritic shafts (Lacey et al., 2007). It is not known whether these neurons represent functionally different subpopulations of Pf-striatal cells.

The cloning of the vesicular glutamate transporters 1 or 2 (vGluT1 or vGluT2) (Fremeau et al., 2001, 2004) and the demonstration that these transporters are differentially expressed in corticostriatal (vGluT1-positive) or thalamostriatal (vGluT2-positive) terminals (but see Barroso-Chinea et al., 2008) have helped with the assessment of the relative prevalence, and the characterization of the synaptic connectivity of corticostriatal and thalamostriatal terminals in rodents and nonhuman primates. These studies showed that 95% of all vGluT1-positive corticostriatal projections terminate on dendritic spines of striatal neurons, and 5% on dendrites in both rodents and primates. This pattern is different from that of vGluT2-containing thalamostriatal boutons. For instance, only 50–65% (depending on the striatal region) of vGluT2-containing terminals contact dendritic spines in monkeys, while the remaining form asymmetric synapses with dendritic shafts (Raju et al., 2008). In rats, as many as 80% of vGluT2-positive terminals form axo-spinous synapses in the striatum (Raju et al., 2006; Lacey et al., 2007). Whether these differences in the proportion of vGluT2 terminals in contact with striatal spines represent a genuine species difference in the microcircuitry of the thalamostriatal system between primates and non-primates remain to be determined.

It is also important to note that the synaptic connectivity of vGluT2-containing terminals differs between the patch/striosome and the matrix compartments of the striatum in rats. While the ratio of axo-spinous and axo-dendritic synapses for vGluT2-immunoreactive terminals is 90:10 in the patch compartment, it is about 55:45 in the matrix (Raju et al., 2006; Figure 1). The fact that the massive thalamostriatal projection from Pf, a predominant source of axo-dendritic glutamatergic synapses, terminates exclusively in the striatal matrix accounts for this difference in the overall synaptology of vGluT2-containing terminals between the two striatal compartments (Herkenham and Pert, 1981; Sadikot et al., 1992b; Raju et al., 2006). Evidence that activity imbalances between the patch/striosome and matrix compartments may be involved in various basal ganglia disorders (Crittenden and Graybiel, 2011) highlights the potential significance of this compartmental segregation of CM/Pf inputs to the mammalian striatum. In contrast to vGluT2, the pattern of synaptic innervation of corticostriatal vGluT1-positive terminals does not differ between patch/striosome and matrix compartments (Raju et al., 2006).

The use of vGluT1 and vGluT2 also allowed the quantification of the relative prevalence of cortical and thalamic terminals in the rat and monkey striatum. In the monkey post-commissural putamen, ~50% of putative glutamatergic terminals (i.e., those forming asymmetric synapses) express vGluT1, ~25% contain vGluT2 and ~25% do not display immunoreactivity for either vGluT1 or vGluT2 (Raju et al., 2008). In rats, the differences in the prevalence of vGluT1 over vGluT2 terminals is not as striking (35% vGluT1 vs. 25% vGluT2 in rats), and the percentage of putative glutamatergic terminals unlabeled for either transporter subtype is higher (~40%) than in monkeys (Kaneko and Fujiyama, 2002; Fujiyama et al., 2004; Lacey et al., 2005; Fujiyama et al., 2006; Huerta-Ocampo et al., 2013). It remains unclear whether the large proportion of putative glutamatergic terminals that are not immunopositive for vGluT1 or vGluT2 are simply cortical and thalamic boutons that express undetectable levels of vGluT1 or vGluT2, whether they express other, yet unidentified, vGluT(s) or whether they are non-glutamatergic.

### Thalamic inputs to direct vs. indirect pathway neurons

The striatum comprises two main populations of output neurons characterized by their differential dopamine receptors and neuropeptides expression (D1/substance P/dynorphin or D2/enkephalin) (Gerfen, 1984). These so-called “direct” and “indirect pathway” MSNs receive synaptic inputs from the thalamus and the cerebral cortex (Somogyi et al., 1981; Hersch et al., 1995; Sidibe and Smith, 1999; Lanciego et al., 2004; Lei et al., 2004, 2013; Huerta-Ocampo et al., 2013). In rats, the proportion of thalamic (vGluT2-positive) or cortical (vGluT1-positive) terminals in contact with direct or indirect pathway MSNs is very similar when considered as a population (Doig et al., 2010; Lei et al., 2013), but the total number of cortical terminals is higher than the number of thalamic boutons in contact with *individual* MSNs (Huerta-Ocampo et al., 2013). Tract-tracing studies in monkeys suggested that afferents from the CM preferentially innervate direct pathway MSNs in the putamen (Sidibe and Smith, 1996). However, because the CM/Pf (or Pf in rodents) has a unique pattern of synaptic connectivity in the striatum compared with other thalamic nuclei (Figures 1, 2), and because axonal tracers labeled only a subset of CM terminals in this study, the potentially preferential innervation of direct pathway neurons needs to be further assessed using vGluT2 or other more general marker of the CM/Pf-striatal system.

The convergence of thalamic and cortical inputs upon single MSNs is consistent with *in vivo* and *in vitro* electrophysiological analyses showing that single direct or indirect pathway neurons respond to both cortical and thalamic stimulation in rodents (Kocsis and Kitai, 1977; Vandermaelen and Kitai, 1980; Ding et al., 2008; Ellender et al., 2011, 2013; Huerta-Ocampo et al., 2013). Recent studies have suggested that thalamic inputs may gate corticostriatal transmission via regulation of striatal cholinergic interneurons, and that this interaction may modulate behavioral switching and attentional set-shifting (Kimura et al., 2004; Ding et al., 2010; Smith et al., 2011; Sciamanna et al., 2012; Bradfield et al., 2013a,b).

### Relationships between thalamic or cortical terminals and dopaminergic or histaminergic afferents

The modulation of excitatory inputs from the cerebral cortex by dopaminergic afferents from the substantia nigra pars compacta is central to our understanding of the functional properties of the basal ganglia. The post-synaptic cortical-induced excitatory responses are modulated by dopamine acting through a wide variety of pre- and/or post-synaptic mechanisms dependent on the type and localization of dopamine receptors and the physiological state of striatal MSNs (Gonon, 1997; Reynolds et al., 2001; Cragg and Rice, 2004; Surmeier et al., 2007; Rice and Cragg, 2008; Ma et al., 2012). Although the regulatory effects of dopamine on thalamic glutamatergic transmission have not been directly assessed, the similarity between the overall pattern of synaptic connectivity of non-CM/Pf thalamic and cortical terminals with MSNs (Moss and Bolam, 2008) suggests that these two pathways may be regulated in the same manner by nigrostriatal dopamine afferents (Figure 1). The dopaminergic modulation of corticostriatal transmission relies in part on the synaptic convergence of dopaminergic and cortical synapses on individual spines of striatal MSNs (Freund et al., 1984; Bolam and Smith, 1990; Smith et al., 1994) and/or pre-synaptic dopamine-mediated regulation of glutamate release from neighboring cortical terminals (Surmeier et al., 2007). Our recent quantitative ultrastructural analyses of rat tissue immunolabelled to reveal both dopaminergic axons and cortical (vGluT1-positive) or thalamic (vGluT2-positive) terminals in rats showed that both glutamatergic systems display the same structural relationships with dopaminergic afferents, i.e., all thalamic and cortical glutamatergic terminals are located within 1 µm of a dopaminergic synapse suggesting that synaptically released and spilled over dopamine may modulate most glutamatergic terminals in the rodent striatum (Arbuthnott et al., 2000; Arbuthnott and Wickens, 2007; Moss and Bolam, 2008; Rice and Cragg, 2008; but see Xu et al., 2012). However, it is unclear whether this general concept of interaction between dopaminergic and thalamostriatal afferents also applies to CM/Pf-striatal terminals that form axo-dendritic synapses. In light of our previous tracing studies which showed that axo-dendritic thalamic inputs from CM and dopaminergic terminals do not display significant structural relationships on the dendritic surface of striatal neurons (Smith et al., 1994), it is likely that the interactions between dopaminergic afferents and CM/Pf or non-CM/Pf thalamostriatal synapses differ (Figure 1).

Glutamatergic inputs from the cerebral cortex and thalamus to both direct and indirect pathway MSNs are also modulated pre-synaptically by histamine (Ellender et al., 2011). Histaminergic projections to the striatum that originate in the hypothalamus negatively modulate corticostriatal and thalamostriatal transmission through histamine H3 receptors.

## Afferent connections of center median/parafascicular (CM/Pf)

In addition to the massive GABAergic projections from the GPi and SNr (see above), the CM receives inputs from motor, premotor and somatosensory cortices (Mehler, 1966; Kuypers and Lawrence, 1967; Kunzle, 1976, 1978; Catsman-Berrevoets and Kuypers, 1978; DeVito and Anderson, 1982), while the Pf is the main target of the frontal and supplementary eye fields (Huerta et al., 1986; Leichnetz and Goldberg, 1988) and associative areas of the parietal cortex (Ipekchyan, 2011). The CM/Pf complex also receives significant afferents from various subcortical sources, including the superior colliculus (Grunwerg et al., 1992; Redgrave et al., 2010), the pedunculopontine tegmental nucleus (Pare et al., 1988; Parent et al., 1988; Barroso-Chinea et al., 2011), the cerebellum (Royce et al., 1991; Ichinohe et al., 2000), the raphe nuclei, the locus coeruleus (Lavoie and Parent, 1991; Royce et al., 1991; Vertes et al., 2010), and from the mesencephalic, pontine and medullary reticular formation (Comans and Snow, 1981; Steriade and Glenn, 1982; Hallanger et al., 1987; Cornwall and Phillipson, 1988; Vertes and Martin, 1988; Royce et al., 1991; Newman and Ginsberg, 1994).

## Physiology of the thalamostriatal projections

### *In vivo* recording studies

Experiments to study the role of the projections from the CM/Pf to the striatum date back to the 1970s. These studies described that electrical stimulation of the intralaminar nuclei induces short-latency excitatory post-synaptic potentials (EPSPs) in anesthetized cats and rats (Kocsis and Kitai, 1977; Vandermaelen and Kitai, 1980), confirming the existence of a direct glutamatergic thalamostriatal connection. Later, Wilson and colleagues demonstrated that these responses occurred in striatal MSNs (Wilson et al., 1983) and in cholinergic interneurons (Wilson et al., 1990). In addition to short-latency EPSPs, both types of neurons also showed prolonged inhibitions, or long-latency excitations (Wilson et al., 1983, 1990), indicating that the thalamic stimulation also engaged polysynaptic pathways.

**Figure 3:**
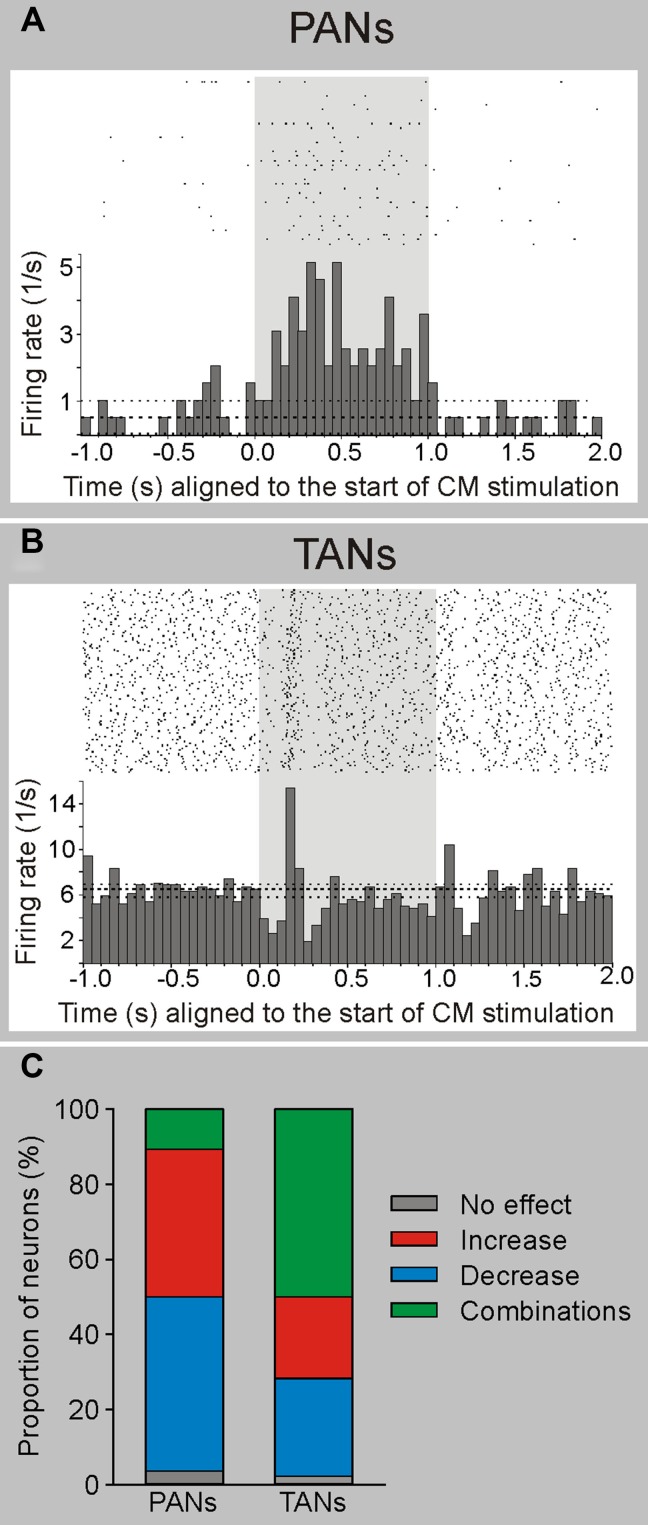
**Electrophysiological responses of striatal neurons to electrical stimulation of CM in awake monkeys.** Electrical stimulation (100 Hz, 100 pulses-shaded area) of CM evokes responses in PANs (putatively MSNs) and TANs (putatively cholinergic interneurons) in awake rhesus monkeys. **(A)** Example of a PAN responding with increased firing to electrical CM stimulation. **(B)** Example of a TAN responding with a brief decrease followed by an increase in firing to CM stimulation. The histograms and rasters are aligned to the start of stimulation trains. **(C)** Summary of responses. The majority of PANs display increases or decreases in firing rate, while most TANs present combinatory (increases and decreases) responses following CM stimulation (see Nanda et al., 2009 for more details).

In more recent studies in awake monkeys, we carried out extracellular recordings in the striatum during electrical stimulation of the CM/Pf complex (Nanda et al., 2009; Figure 3). We found that striatal cells did not respond to single-pulse stimulation, but that many neurons showed long-latency (tens of milliseconds) responses to burst stimulation (100 Hz, 1 s) of the CM/Pf. While phasically active neurons (PANs), which likely correspond to striatal MSNs, responded mainly with increases in firing, tonically active striatal neurons (TANs, likely to be cholinergic interneurons) often showed combinations of increases and decreases in firing (Nanda et al., 2009; Figure 3). Both types of responses were most likely generated by activation of the intrastriatal circuitry. As mentioned above, anatomical observations support the idea that CM/Pf terminals contact both striatal GABAergic and cholinergic elements in primates and non-primates (Sidibe and Smith, 1999), and that cholinergic interneurons receive GABAergic synaptic inputs from collaterals of direct and indirect pathway MSNs (Gonzales et al., 2013). These collaterals may mediate the decreases in firing after the activation of the presumably excitatory thalamostriatal projections.

The eventual effects of CM stimulation on the striatal circuitry may depend on the experimental conditions chosen. For instance, pharmacological stimulation of the Pf in rats, or electrical stimulation of the CM/Pf in monkeys, was shown to reduce striatal acetylcholine levels, an effect that can be reversed by intrastriatal administration of GABA_A_ receptor antagonists (Zackheim and Abercrombie, 2005; Nanda et al., 2009). The reduction in acetylcholine levels could be explained assuming that the thalamic activation drives intrastriatal GABAergic neurons that then secondarily inhibit cholinergic interneurons. However, other studies showed that electrical stimulation of Pf increased the level of acetylcholine in the rat striatum (Consolo et al., 1996a) in an NMDA-receptor dependent manner.

### *In vitro* recordings in brain slices

A new rat brain slice preparation that partly preserved thalamostriatal axons (Smeal et al., 2007) has enabled studies of the chemical and functional properties of thalamostriatal synapses and the potential relationships between thalamostriatal and corticostriatal systems in normal state (Ding et al., 2008; Smeal et al., 2008). Using this preparation, the ratio of NMDA/non-NMDA glutamatergic receptors was found to be higher at thalamic than cortical synapses (Ding et al., 2008; Smeal et al., 2008), an observation that extends earlier neurochemical studies in adult rats (Baldi et al., 1995; Consolo et al., 1996a,b). This slice preparation has also lead to additional data suggesting that the thalamostriatal system gates corticostriatal signaling via activation of striatal cholinergic interneurons, and that this functional interaction might be altered in mouse model of dystonia (Ding et al., 2008; Sciamanna et al., 2012). However, it is a limitation of this preparation that thalamostriatal projections from CM/Pf cannot be distinguished from those originating in other parts of the thalamus.

**Figure 4:**
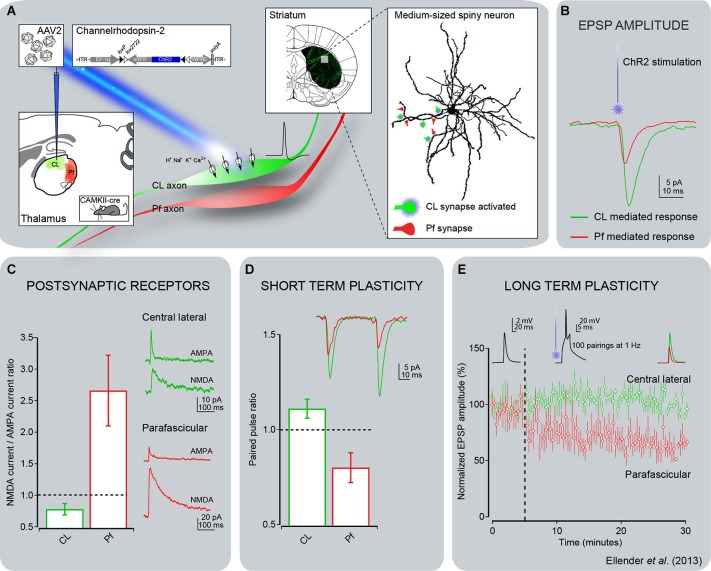
**Electrophysiological responses of striatal MSNs to optogenetic activation of thalamostriatal terminals from CL or Pf in mice. (A)** Channelrhodopsin-2 (ChR2) was delivered to either the CL or Pf thalamic nucleus using a stereotaxic injection of adeno-associated virus containing the double-floxed sequence for ChR2-YFP in CAMKII-cre mice. This approach enabled expression of ChR2 in the excitatory thalamic neurons of either the CL or Pf nucleus including in their axonal arbor. Thalamic axons expressing ChR2-YFP were readily visible in acute striatal slices and this method allowed for activation of only those synapses originating from neurons from the injected thalamic nucleus by illumination of these slices with blue (473 nm) laser or LED light. **(B)** Whole-cell patch-clamp recordings of striatal MSNs showed that activation of CL synapses led to consistently larger post-synaptic responses than activation of Pf synapses. **(C)** Detailed investigation of the glutamate receptor-mediated currents revealed the CL synapses exhibit predominantly AMPA receptor-mediated currents in response to light activation and the Pf synapses exhibit predominantly NMDA receptor-mediated currents. **(D)** The short term plastic properties of these synapses were investigated by repetitive activation of the synapses in close succession. This revealed that inputs from CL exhibit short term facilitation, in which the response to the second activation has a larger amplitude than the response to the first activation, whereas inputs from Pf exhibit short term depression. **(E)** The long term plastic properties of these synapses were investigated using a spike timing-dependent plasticity protocol, consisting of the pairing of optical activation of a pre-synaptic thalamic input with a post-synaptic action potential in a MSN. This protocol induced a clear long term depression of synaptic efficacy at Pf synapses, but no plasticity was observed at CL synapses. The same observation was made using either a pre-post or post-pre pairing, with only the pre-post pairing shown for clarity (see Ellender et al., 2013 for more details).

The introduction of optogenetic methods helped to further characterize the properties of specific thalamostriatal synapses in rats (Ellender et al., 2013). Thus, neurons in the central lateral nucleus (CL) have bushy, frequently branching, dendrites and, under anesthesia, fire action potentials in the form of low-threshold Ca^2+^ spike bursts, while Pf neurons have long, infrequently branching dendrites and give rise to action potentials that are only rarely in the form of low-threshold bursts (Lacey et al., 2007; Figure 2). In the striatum, thalamostriatal terminals from the CL terminate almost exclusively on dendritic spines, while Pf boutons target predominantly dendritic shafts (Figure 2). *In vitro* optogenetic activation of the different pathways combined with whole-cell, patch-clamp recordings of direct or indirect pathway MSNs in adult mice (Ellender et al., 2013) revealed that stimulation of CL synapses leads to large amplitude, predominantly AMPA-receptor mediated, excitatory responses that display short-term facilitation. In contrast, stimulation of Pf synapses gives rise to small amplitude responses that display short-term depression and are largely mediated by post-synaptic NMDA receptors (Ellender et al., 2013; Figure 4). The high frequency Ca^2+^ spike bursts in CL neurons together with the synaptic properties of CL thalamostriatal synapses suggests that thalamic inputs from CL are well suited to driving MSNs to depolarization and hence firing (Ellender et al., 2013). In contrast, the firing characteristics and properties of Pf neurons and their thalamostriatal synapses suggest that these are better suited to exert modulatory effects on striatal MSNs, which could be in the form of facilitating Ca^2+^-dependent processes. Furthermore, pairing Pf pre-synaptic stimulation with action potentials in MSNs leads to NMDA receptor- and Ca^2+^-dependent long-term depression at these synapses (Ellender et al., 2013; Figure 4).

## The role of the center median/parafascicular (CM/Pf) thalamostriatal system in cognition

The CM/Pf-striatal system is now thought to be critical in mediating basal ganglia responses to attention-related stimuli, and may be engaged in behavioral switching and reinforcement functions (Kimura et al., 2004; Minamimoto et al., 2009; Smith et al., 2011; Bradfield et al., 2013a).

### Responses of center median/parafascicular (CM/Pf) neurons in attention-related tasks

**Figure 5:**
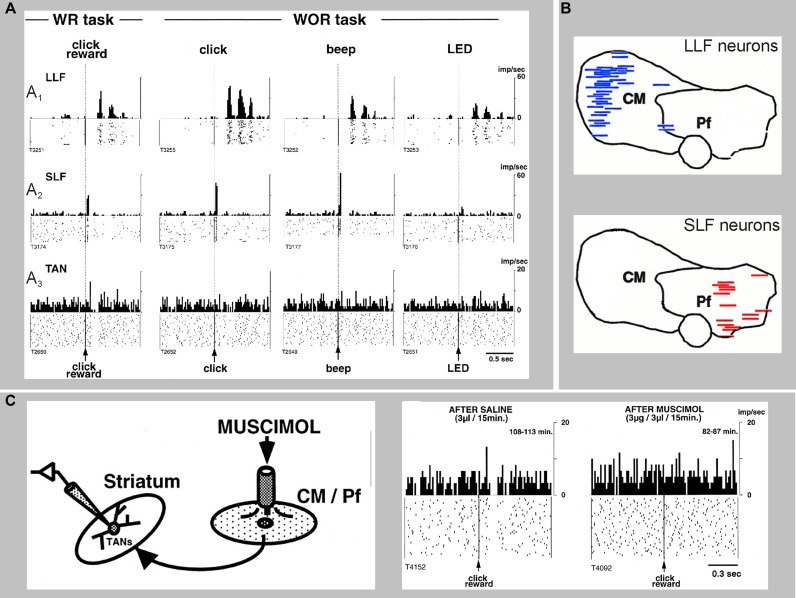
**Sensory responses of two types of CM/Pf neurons, and a striatal TAN recorded during the presentation of a stimulus with or without reward (WR vs. WOR).** The spike rasters and histograms are aligned to the time of presentation of the stimulus. **(A_1_)** Representative activity of a CM neuron with long-latency facilitation (LLF) following stimulus presentation. **(A_2_)** Activity of a Pf neuron showing short-latency facilitation (SLF) after stimulus. **(A_3_)** Activity of a TAN. Note that thalamic responses occur in both WR and WOR tasks, whereas TAN responses occur only in the WR task. **(B)** Localization of LLF and SLF neurons in the monkey CM/Pf complex. Note the complete segregation of these two populations of neurons in CM or Pf. **(C)** Application of the GABA_A_ receptor agonist muscimol in CM/Pf alters the pattern of responses of striatal TANs to the presentation of reward-related sensory stimuli in awake monkeys, The rasters and histograms on the right demonstrate that the pauses in the TAN responses are abolished after application of muscimol in CM/Pf (reproduced with permission from Matsumoto et al., 2001).

Because the intralaminar nuclei receive massive ascending projections from the reticular formation and various brainstem regions (see above), and have long been known as the source(s) of widely distributed “nonspecific” thalamocortical projections, these thalamic nuclei are considered part of the ascending “reticular activating system” that regulates arousal and attention (as reviewed in Van der Werf et al., 2002). In line with this concept, functional imaging studies in humans demonstrated a significant increase of activity in CM/Pf during processing of attention-related stimuli (Kinomura et al., 1996; Hulme et al., 2010; Metzger et al., 2010). More recent observations in primates showed that CM and Pf neurons respond to behaviorally salient visual, auditory and somatosensory stimuli (Matsumoto et al., 2001; Minamimoto and Kimura, 2002; Minamimoto et al., 2005; Figure 5). In these studies, the response latencies of Pf neurons were much shorter than those of CM neurons (Figure 5). Compatible with the view that responses of CM/Pf neurons to external events are related to attention, the initially vigorous responses fade quickly upon repeated stimulus presentation if stimuli were not followed by reward, and, thus, lose their salience (Matsumoto et al., 2001; Minamimoto and Kimura, 2002; Kimura et al., 2004; Minamimoto et al., 2005). Acute pharmacological inactivation of Pf in monkeys disrupts attention processing more efficiently than CM inactivation (Minamimoto and Kimura, 2002). The functional responses of CM/Pf neurons to attention-related stimuli thus suggest a role of the CM/Pf-striatal system in cognition, most particularly related to attention shifting, behavior switching and reinforcement processes (Matsumoto et al., 2001; Minamimoto and Kimura, 2002; Kimura et al., 2004; Minamimoto et al., 2005; Smith et al., 2011; Bradfield et al., 2013a,b). There is also evidence that sensory-responsive CM neurons may be involved in mechanisms needed for decision-making and biasing actions (Minamimoto et al., 2005).

Additional evidence for a “cognitive” role of the CM/Pf projections to the striatum comes from studies in mice in which selective immunotoxin lesions or pharmacological inactivation of the Pf-striatal projection, impair performance in a discrimination learning task (Brown et al., 2010; Kato et al., 2011). Furthermore, recent evidence indicates the Pf projection to the posterior dorsomedial striatum is involved in regulating the interaction between new and previously learned stimuli (Bradfield et al., 2013a,b). It is noteworthy that neither lesion nor pharmacological inactivation of CM/Pf in monkeys or rodents lead to motor impairments (Matsumoto et al., 2001; Minamimoto and Kimura, 2002; Brown et al., 2010; Kato et al., 2011).

### Center median/parafascicular (CM/Pf)-striatal system regulation of striatal cholinergic interneurons

Reward-associated events evoke pause responses in striatal TANs (which are likely to be cholinergic interneurons) (Goldberg and Reynolds, 2011). These responses are regulated in part by the CM/Pf-striatal system, because they are almost completely abolished by chemical inactivation of the CM/Pf complex in monkeys (Matsumoto et al., 2001; Figure 5). Furthermore, the removal of Pf inputs to cholinergic interneurons reduces the firing rate of these neurons and produces an enduring deficit in goal-directed learning (Bradfield et al., 2013a). These observations are consistent with the fact that cholinergic interneurons receive synaptic inputs from CM/Pf (Lapper and Bolam, 1992; Sidibe and Smith, 1999), that CM stimulation strongly affects TAN activity patterns (Wilson et al., 1990; Nanda et al., 2009) and that CM/Pf alterations affect striatal acetylcholine release (Consolo et al., 1996a,b; Zackheim and Abercrombie, 2005; Nanda et al., 2009). As mentioned above, several mechanisms have been proposed to explain how activation of the glutamatergic CM/Pf-striatal projection evokes inhibitions or pauses in TAN firing, including the involvement of intercalated GABAergic and dopaminergic elements, as well as intrinsic properties of cholinergic interneurons (Ding et al., 2010; Goldberg and Reynolds, 2011; Sciamanna et al., 2012; Threlfell et al., 2012).

## Degeneration of the thalamostriatal system in Parkinson’s disease (PD)

**Figure 6:**
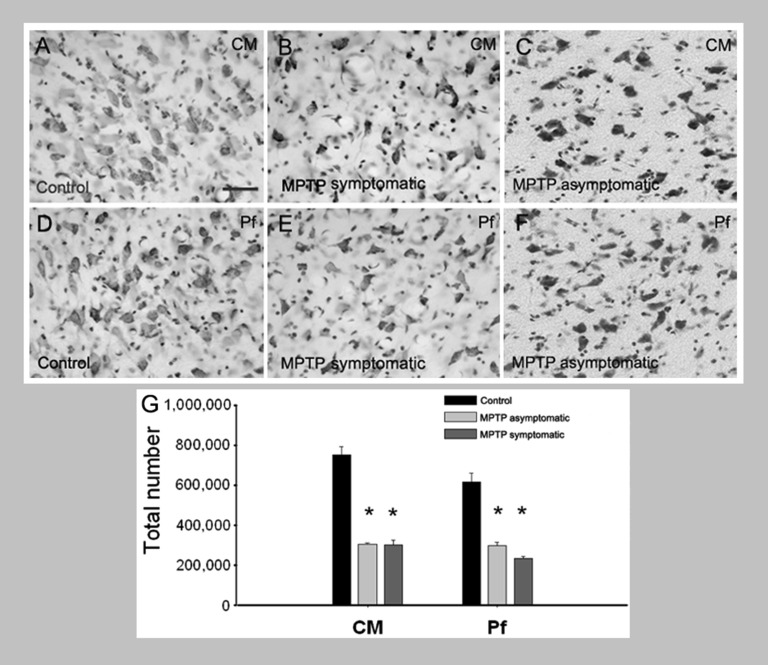
**Loss of CM/Pf neurons in MPTP-treated monkeys. (A–G)** Light micrographs of neurons in CM or Pf of control monkeys **(A, D)**, MPTP-treated motor symptomatic **(B, E)** and MPTP-treated motor asymptomatic **(C, F)** animals. The motor asymptomatic animals had ~40% striatal dopamine loss and did not display any Parkinsonian motor symptoms, while the motor symptomatic monkeys had more than 80% striatal dopamine loss and displayed moderate to severe Parkinsonism. Note the significant reduction in neuronal density in CM and Pf of both symptomatic and asymptomatic MPTP-treated monkeys. **(G)** Stereological cell counts demonstrate a significant loss of neurons in both CM and Pf of the two groups of MPTP-treated monkeys compared with controls.

Postmortem studies have shown that 30–40% of CM/Pf neurons are lost in PD patients with mild motor deficits, and that the extent of CM/Pf degeneration does not further progress with the severity of the Parkinsonian motor signs (Xuereb et al., 1991; Heinsen et al., 1996; Henderson et al., 2000a,b,^2005^; Brooks and Halliday, 2009; Halliday, 2009). We have recently found a similarly robust loss of CM/Pf neurons in monkeys that were chronically treated with low doses of the neurotoxin 1-methyl-4-phenyl-1,2,3,6-tetrahydropyridine (MPTP), even in motor asymptomatic animals with minimal nigrostriatal dopaminergic denervation (Villalba et al., 2014; Figure 6). In PD patients and MPTP-treated monkeys, this thalamic degeneration predominantly affects CM/Pf (Henderson et al., 2000a,b; Villalba et al., 2014), although significant neuronal loss was also reported in the parataenial, cucullar and central lateral nuclei of PD patients (Halliday, 2009). In the brain of PD patients, α-synuclein deposition was found in the latter three nuclei, but not as much in CM/Pf (Brooks and Halliday, 2009). Parvalbumin-negative neurons are particularly affected in CM (Halliday, 2009). It is noteworthy that robust CM/Pf neuronal loss is not only found in PD, but has also been found in other neurodegenerative diseases, including progressive supranuclear palsy and Huntington’s disease (Heinsen et al., 1996; Henderson et al., 2000a,b). The cellular properties of CM/Pf neurons needs to be studied in more detail to determine the potential factors that make them more sensitive to degeneration than other thalamic cells in these diseases (see also below).

In rodent and primate models of PD, striatal dopamine loss is associated with a loss of glutamatergic synapses (Ingham et al., 1998; Villalba et al., 2013), which is consistent with a loss of spines on striatal MSNs in PD postmortem material (see below). Stereological estimates of the number of glutamatergic synapses formed by vGluT1- (marker of cortical terminals) or vGluT2- (marker of thalamic terminals) containing terminals in the putamen of MPTP-treated monkeys showed that the number of vGluT2-positive terminals, but not that of vGluT1-containing boutons, is substantially reduced in Parkinsonian animals (Villalba et al., 2013). This suggests that the loss of thalamic glutamatergic inputs to the striatum outweighs the loss of cortical terminals in MPTP-treated monkeys (Villalba et al., 2013). Because CM/Pf inputs to striatal MSNs predominantly terminate on dendritic shafts (see above), these findings are in line with our previous studies which had demonstrated that MPTP treatment reduces the relative prevalence of vGluT2-positive axo-dendritic synapses in the putamen more strongly than that of axo-spinous synapses (Raju et al., 2008). Together, these results support the hypothesis that the loss of CM/Pf inputs to the striatum prominently contributes to the glutamatergic deafferentation of MSNs in PD. It is not yet clear whether the loss of striatal inputs from CM/Pf also affects the glutamatergic innervation of cholinergic interneurons.

The loss of glutamatergic afferents is reflected in a profound loss of dendritic spines of striatal MSNs in PD patients (Stephens et al., 2005; Zaja-Milatovic et al., 2005), rodent models of PD (Ingham et al., 1989; Day et al., 2006; Kusnoor et al., 2009) and in MPTP-treated monkeys (Raju et al., 2008; Smith et al., 2009; Villalba et al., 2009, 2013; Villalba and Smith, 2010, 2011, 2013). In the latter studies, a 40–50% spine loss was found in the sensorimotor striatum, similar to findings in PD patients (Zaja-Milatovic et al., 2005; Villalba et al., 2009). The link between the aforementioned striatal glutamatergic deafferentation and spine loss remains uncertain. For instance, it remains unclear whether the CM/Pf degeneration and the resulting loss of glutamatergic inputs to the striatum contribute to the changes in the number and morphology of dendritic spines in PD. As a further complication, the use of dopaminergic drugs can also affect spine growth and morphology. Thus, a recent study showed aberrant restoration of axo-spinous synapses that affect corticostriatal, but not thalamostriatal synapses, in unilaterally 6-hydroxydopamine (OHDA)-lesioned rats that developed L-3,4-didroxyphenylalanine (L-DOPA)-induced dyskinesias (Zhang et al., 2013), suggesting a differential impairment of the two glutamatergic systems in dyskinesia. Details about the extent of Pf neuronal loss in these animals are needed to translate these findings to human PD patients with L-DOPA-induced dyskinesia.

### Potential reasons for center median/parafascicular (CM/Pf) neuron loss in PD

Although it remains unclear why CM/Pf neurons are particularly sensitive to neurodegeneration in PD and other disorders (Halliday et al., 2005; Halliday, 2009), their chemical phenotype and sensitivity to chronic MPTP administration in non-human primates (Villalba et al., 2014) may provide clues. The CM/Pf nuclei do not carry a specifically high level of α-synuclein deposits in PD patients (Brooks and Halliday, 2009), so that their vulnerability in PD cannot be explained by the burden of α-synuclein aggregation.

In rodents, striatal terminals from the Pf express immunoreactivity for the protein cerebellin 1, a neurochemical feature that appears to be specific for the CM/Pf-striatal system, and may also regulate the morphology of striatal spines (Kusnoor et al., 2010). It remains to be established whether this unique molecular characteristic amongst thalamic neurons contributes to their susceptibility. It is also possible that the absence of calcium binding proteins expression in CM/Pf neurons determines their differential vulnerability. In humans, postmortem studies have shown that subpopulations of parvalbumin-containing neurons are mainly affected in Pf, while non-parvalbumin/non-calbindin neurons are more specifically targeted in CM (Henderson et al., 2000a). In MPTP-treated animals, the degeneration of CM/Pf could instead be due to direct toxic effects of the MPTP metabolite, 1-methyl-4-phenylpyridinium (MPP+), on the thalamus, independent of its effects on the nigrostriatal system (Villalba et al., 2014). Consistent with this possibility, injections of MPP+ into the rodent striatum resulted in a major neuronal loss in Pf, without significant effects on cortical neurons (Ghorayeb et al., 2002).

## The center median/parafascicular (CM/Pf) as a target of neurosurgical interventions in brain disorders

Although the physiological properties of the caudal intralaminar nuclei and their projections remain poorly characterized, these nuclei have been used as targets for surgical interventions, aimed at treating pain, seizures, impairments of consciousness, or movement disorders. We will focus our discussion on the use of neurosurgical procedures as treatment of movement disorders, because this use is most easily linked to the interactions between CM/Pf and the basal ganglia. These procedures have been used specifically in patients with disabling TS, or with PD. The mechanisms of action of CM/Pf interventions in these diseases, and the specifics of the optimal surgical approach and DBS characteristics remain matters of speculation. Furthermore, inclusion and exclusion criteria for trials of these interventions are only beginning to emerge for TS patients, while no formal criteria have yet been developed for trials in patients with PD.

### Ablative surgeries of center median/parafascicular (CM/Pf)

Since the 1960s, unilateral or bilateral lesions of the intralaminar and medial thalamic nuclei, as well as the nucleus ventro-oralis internus (Voi) have been empirically used to treat patients with TS (see below) (Hassler and Dieckmann, 1970, 1973; de Divitiis et al., 1977; Hassler, 1982). These studies have reported impressive reductions in tic frequency in these patients, along with a lesser reversal of compulsive symptoms. The effects of CM/Pf lesions in other movement disorders (such as Parkinsonism) have not been extensively characterized. However, in rodents, Pf lesions were shown to prevent the neurochemical changes produced by dopamine denervation in different basal ganglia nuclei (Kerkerian-Le Goff et al., 2009). Similar experiments in MPTP-treated primates have not resulted in significant anti-parkinsonian effects (Lanciego et al., 2008).

### CM/Pf deep brain stimulation and TS

TS is a neuropsychiatric disorder of childhood onset. Patients develop rapid, stereotyped movements (tics) which typically peak in preadolescence and decline in the later teenage years. Many TS patients also suffer from psychiatric comorbidities, such as obsessive compulsive disorder, attention-deficit hyperactivity disorder, or depression. Most patients are successfully (albeit partially) treated with neuroleptics and other drugs or with behavioral therapy. However, a few patients continue to experience severe tics in adulthood. These patients are candidates for neurosurgical procedures. Although still under considerable debate, TS may be the result of abnormal GABAergic or dopaminergic transmission in the basal ganglia (see, e.g., Buse et al., 2013; Worbe et al., 2013), involving both motor and limbic basal ganglia-thalamocortical circuitry.

Although there is little evidence linking the pathology or functional disturbances of CM/Pf to TS, this nuclear complex has been a major focus of surgical treatment of this condition, mostly because of the early empirical evidence with ablative treatments (see above). Early investigations of the use of stimulation of CM/Pf in movement disorders were carried out in patients that were enrolled in pain treatment studies, but also suffered from movement disorders (Andy, 1980; Krauss et al., 2002). Significant symptomatic improvements of motor dysfunctions were reported in these studies, but the stimulation parameters were not communicated. Since then, impressive reductions in tic frequency and severity, perhaps with greater effectiveness against motor than vocal tics, have been reported, although the number of TS patients treated with CM/Pf DBS remains small (Visser-Vandewalle et al., 2003, 2004, 2006; Temel and Visser-Vandewalle, 2004; Houeto et al., 2005; Ackermans et al., 2006, 2008, 2010, 2011; Bajwa et al., 2007; Maciunas et al., 2007; Servello et al., 2008, 2010; Shields et al., 2008; Porta et al., 2009; Hariz and Robertson, 2010; Sassi et al., 2011; Maling et al., 2012; Savica et al., 2012; Visser-Vandewalle and Kuhn, 2013). The time course of tic improvement varies between individuals, ranging from immediate effects (Visser-Vandewalle et al., 2003; Maciunas et al., 2007) to a more protracted time course (Maciunas et al., 2007; Servello et al., 2008). In addition to the motor symptoms of the disease, CM/Pf DBS also effectively alleviated some of the psychiatric components of TS, including obsessive-compulsive behaviors and anxiety (Houeto et al., 2005; Mink, 2006; Visser-Vandewalle et al., 2006; Neuner et al., 2009; Krack et al., 2010; Sassi et al., 2011). The mechanisms of action of CM/Pf stimulation on TS signs and symptoms remain unclear, but the anatomy and potential role of the thalamostriatal system from CM/Pf in cognition, as well as functional studies indicating the key role of CM/Pf in regulating striatal cholinergic interneurons activity (see above) in animals, suggest that CM/Pf stimulation may mediate its effects through complex regulation of striatal microcircuits that influence both motor and non-motor basal ganglia-thalamocortical and thalamostriatal networks (Nanda et al., 2009; Kim et al., 2013).

### Center median/parafascicular (CM/Pf) DBS and Parkinson’s disease (PD)

CM/Pf DBS was found to provide significant anti-parkinsonian benefits in 6-OHDA-treated rats (Jouve et al., 2010). CM/Pf DBS has also been used in a few PD patients. These studies have suggested that CM/Pf DBS may have anti-dyskinetic effects and reduce freezing of gait, a symptom that is not satisfactorily treated with either medications or conventional DBS approaches directed at subthalamic and pallidal targets (Caparros-Lefebvre et al., 1999; Mazzone et al., 2006). More recent studies have suggested that CM/Pf DBS may also reduce Parkinsonian tremor (Peppe et al., 2008; Stefani et al., 2009).

## Concluding remarks and future studies

Despite significant progress in our understanding of the anatomy, physiology and pathophysiology of the thalamostriatal systems, many unresolved issues remain. The cloning of vGluT1 and vGluT2 has had a significant impact in our understanding of the anatomical and synaptic organization of the thalamostriatal systems, allowing us to further appreciate that the thalamus is a massive source of extrinsic glutamatergic inputs to the striatum that originates either in the CM/Pf nuclear complex, or in the numerous non-CM/Pf thalamic nuclei. Future studies aimed at understanding the physiological role of these multiple thalamostriatal circuits, and their functional interactions with the corticostriatal and nigrostriatal systems, are warranted to decipher the mechanisms by which these extrinsic afferents regulate basal ganglia functions.

Because of the complex relationships between the cerebral cortex, thalamus and striatum, the use of traditional stimulation or lesion methods has had a limited impact in our understanding of the physiology and synaptic properties of thalamostriatal connections. However, as presented in this review, optogenetic approaches help overcome some of these technical challenges, setting the stage for a rigorous and detailed characterization of the physiology and pathophysiology of the CM/Pf vs. non-CM/Pf-striatal systems in normal and diseased states.

The major breakthroughs that were recently made in characterizing some aspects of the role of the CM/Pf-striatal system in regulating the physiological responses of striatal cholinergic interneurons to attention-related salient sensory stimuli provide a deeper understanding of the mechanisms involved by which the basal ganglia regulate activities such as behavioral switching, attentional set-shifting and reinforcement. Because the CM/Pf-striatal system undergoes massive degeneration in PD, future studies aimed at assessing the effects of this degeneration upon attention and other basal ganglia-related cognitive functions are needed. A better understanding of the respective role played by the thalamostriatal vs. the nigrostriatal dopamine systems in the regulation of cholinergic interneuron activity is another area of great interest for future studies.

In light of neuropathology studies of the thalamus in human patients with PD (Halliday, 2009), combined with studies in MPTP-treated non-human primates, it appears that CM/Pf neurons are particularly sensitive to degeneration in PD, neurodegenerative diseases, and neurotoxic insults. Thus, future studies aimed at elucidating the chemical, physiological and pharmacological properties of CM/Pf neurons vs. other thalamic cells are essential to determine the basis for the selective vulnerability of CM/Pf neurons in brain disorders.

Finally, another area of great interest is the use of DBS of CM/Pf in movement disorders, most particularly in TS. We need to understand better how stimulation of the thalamostriatal system from CM/Pf alleviates tics and psychiatric symptoms of this disease. Furthermore, rigorous blinded trials in a large number of patients still need to be done before this treatment can be recommended for patients with TS.

## Conflict of interest statement

The authors declare that the research was conducted in the absence of any commercial or financial relationships that could be construed as a potential conflict of interest.
